# No Effect of Anodal Transcranial Direct Current Stimulation (tDCS) Over hMT+ on Motion Perception Learning

**DOI:** 10.3389/fnins.2018.01044

**Published:** 2019-01-17

**Authors:** Stephanie J. Larcombe, Christopher Kennard, Jacinta O’Shea, Holly Bridge

**Affiliations:** ^1^Nuffield Department of Clinical Neurosciences, University of Oxford, Oxford, United Kingdom; ^2^Wellcome Centre for Integrative Neuroimaging – Oxford Centre for Functional MRI of the Brain, Nuffield Department of Clinical Neurosciences, University of Oxford, Oxford, United Kingdom

**Keywords:** transcranial direct current stimulation, brain stimulation, motion perception, perceptual learning, visual area hMT+

## Abstract

**Background:** Human visual cortical area hMT+, like its homolog MT in the macaque monkey, has been shown to be particularly selective to visual motion. After damage to the primary visual cortex (V1), patients often exhibit preserved ability to detect moving stimuli, which is associated with neural activity in area hMT+. As an anatomical substrate that underlies residual function in the absence of V1, promoting functional plasticity within hMT+ could potentially boost visual performance despite primary visual cortical damage.

**Objective:** To establish in healthy participants whether it is possible to use transcranial direct current stimulation (tDCS) over hMT+ to potentiate learning of visual motion direction discrimination.

**Methods:** Twenty-one participants were trained daily for 5 days on a visual motion direction discrimination task. Task difficulty was increased as performance improved, by decreasing the proportion of coherently moving dots, such that participants were always performing at psychophysical threshold. tDCS, either anodal or sham, was applied daily during 20 min of training. Task performance was assessed at baseline and at the end of the training period. Performance was also compared with a third group of 10 participants from an earlier study who had undergone the same procedures but without tDCS.

**Results:** All participants showed improved task performance both during and after training. Contrary to our hypothesis, anodal tDCS did not further improve performance compared to sham stimulation or no stimulation. Bayesian statistics indicated weak evidence in favor of the null hypothesis.

**Conclusion:** This study found no evidence for a robust effect of anodal tDCS over hMT+ on visual motion direction discrimination learning in the young healthy visual system, although more subtle effects may have been missed in the relatively small sample size.

## Introduction

The principal pathway conveying visual information from the eye to the brain projects via the primary visual cortex (V1), the largest cortical visual area. The critical role of this area in vision is reflected in the fact that any damage to this region can lead to cortical blindness. However, even after damage to V1, many patients continue to show brain activity in the cortical motion area human MT+ (hMT+) (Zeki and Ffytche, 1998; Morland et al., 2004; Bridge et al., 2010; Ajina et al., 2015) and some are adept at detecting moving stimuli, a capacity known as blindsight (Cowey, 2010). Hence area hMT+ is a potential intervention target for rehabilitation regimes that aim to improve visual function after V1 damage (Das and Huxlin, 2010; Ajina and Bridge, 2016).

In the healthy visual system, the specialized role of hMT+ in humans and middle temporal area (MT) in the non-human primate has been demonstrated using multiple techniques, including electrophysiology (Britten et al., 1992, 1993; Shadlen and Newsome, 1996), lesion studies (Zihl et al., 1983; Newsome et al., 1985; Newsome and Pare, 1988), functional magnetic resonance imaging (fMRI) (Tootell et al., 1995) and electrical stimulation (Salzman et al., 1992). Given this role it could be hypothesized that perceptual training on motion discrimination should result in functional changes within MT. However, this does not appear to be the case, at least in the macaque. Law and Gold (2008, 2009, 2010) have shown that learning a motion task does not change neuronal properties in MT, but rather this occurs at the level of the sensory-motor decision, in lateral intraparietal area (LIP). Nevertheless, Liu and Pack (2017) demonstrated that while training on a motion discrimination task did not change the sensitivity of individual MT neurons, after training there was an increased effect of MT microstimulation on biasing motion direction decisions.

In humans, learning a visual motion discrimination task over 5 days causes an increase in neural activity in MST, part of the human motion complex, which correlates with the amount of learning (Larcombe et al., 2017a), suggesting a functional role for MST in the improved performance. Since this region often remains active in patients who have suffered damage to V1, it may be that visual discrimination training could strengthen subcortical connections to visual motion areas and increase residual visual function. While boosting performance with training is beneficial, addition of an adjunct intervention to increase plasticity, such as pharmacological enhancement of acetylcholine levels (Rokem and Silver, 2010), can further potentiate the effect.

Here, we tested whether a different neuroplasticity intervention, non-invasive brain stimulation over hMT+, when applied during training, could also increase learning. We chose to stimulate using anodal transcranial direct current stimulation (tDCS) and compare this to sham. Anodal tDCS has been shown to increase visual cortical excitability (Antal et al., 2003a,b), while anodal tDCS to both V1 and hMT+ has been shown to enhance visual functioning (Antal et al., 2004a; Kraft et al., 2010; Olma et al., 2011; Sczesny-Kaiser et al., 2016). In particular, several studies have stimulated hMT+ and found an improvement in motion direction perception over a single experimental session (Antal et al., 2004a; Battaglini et al., 2017), suggesting the potential for increased plasticity with tDCS. In the motor system, anodal tDCS applied to primary motor cortex during training has been shown to enhance acquisition and consolidation of visuomotor learning (Reis et al., 2009; O’Shea et al., 2017). The current study tested whether anodal tDCS of hMT+ would augment learning of visual motion direction discrimination.

## Materials and Methods

### Participants

Twenty-four participants (13 female and 11 male; mean = 24.7 years; *SD* = 5.8 years) were randomly assigned to an anodal (*n* = 13) or sham (*n* = 11) stimulation group. Prior to study initiation, participant identifiers from 001 to 024 were allocated pseudorandomly using matlab (by an author not involved in the data acquisition) to either anodal or sham stimulation, to ensure equal distribution of participants between groups. The identifiers were then assigned in sequential order to each participant on entry to the study. Before study completion, three participants withdrew from the study, two from the anodal group and one from the sham group. Owing to incomplete data, these participants were excluded from all analyses. Given the null effect of tDCS, an additional *post hoc* comparison was made against a group of 10 participants from an earlier study who had undergone the same training protocol, but without any stimulation (5 female and 5 male; mean = 23.2 years; *SD* = 3.0 years) (Larcombe et al., 2017b). All participants from the previous study who followed the identical protocol as in the present study were included (the remaining participants had trained either on a different task or for a different duration). The purpose of this *post hoc* contrast was to determine if task performance by participants in the present tDCS study was within the range of normal behavior observed previously without any tDCS.

The study was approved by the local InterDivisional Research Ethics Committee (IDREC) at the University of Oxford (reference MSD-IDREC-C2-2014-025) and all participants gave written, informed consent. Research was carried out in accordance with the Code of Ethics of the World Medical Association (Declaration of Helsinki). All participants underwent safety screening to exclude contraindications to brain stimulation prior to each test day.

### Sample Size

Twenty-four participants were recruited for the study as this is comparable to several tDCS studies in the visual system that found significant effects (Antal et al., 2004a,b) although it is smaller than a more recent study (*n* = 15) (Battaglini et al., 2017). A large effect size would be required to be evident in this population, but the study was designed to test for a large effect of stimulation on learning, as has been reported for anodal tDCS to motor cortex during visuomotor learning (Reis et al., 2009; O’Shea et al., 2017). In addition, the sample size was limited by practical constraints. Since the protocol involved 5 consecutive days of training combined with sham or anodal stimulation, plus pre- and post-training assessment sessions, the 21 complete datasets took 6-months to acquire.

### Visual Task

Participants completed a motion perception task where the instructions were to discriminate the direction of coherently moving dots presented amongst randomly moving distractor dots. Moving dots (*n* = 143) were presented within a circular area 11° in diameter, offset 10° to the left or right of fixation. Dots were high contrast white dots on a black background. The luminance and chromaticity measures (SpectraScan PR-650) were white: 96.8 cd/m^2^ (*x* = 0.289, *y* = 0.312), and black: 0.92 cd/m^2^ (*x* = 0.236, *y* = 0.247). The dot diameter was 0.15°, and the dots moved with a speed of 6°/s for a limited lifetime of 200 ms (12 frames), at a density of 1.5 dots/degree^2^. Dots were born or reborn at random, non-overlapping locations within the stimulus aperture. Coherent motion direction was variable, but restricted to within a 90° angle centered around the horizontal meridian.

**FIGURE 1:**
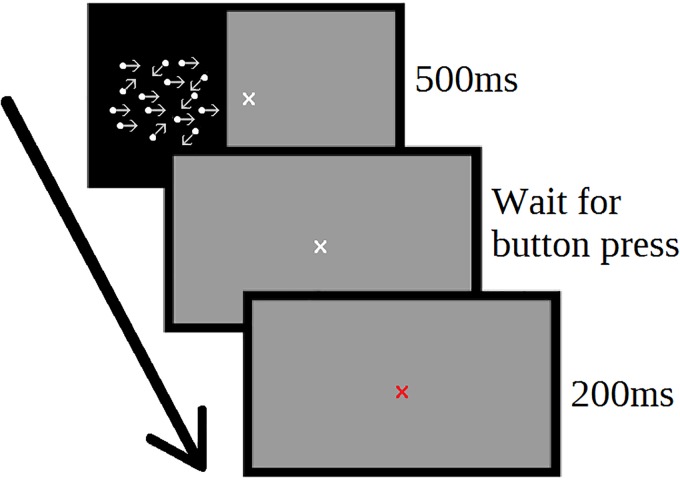
Motion direction discrimination task. Participants determined the direction of coherent motion of moving dots. Each trial consisted of 500 ms stimulus period, followed by an untimed user response window. Following participant response, feedback was provided (red or green fixation cross) for 200 ms, and then the next trial start immediately.

Each trial consisted of a 500 ms stimulus window, a pause for the participant response, and a 200 ms feedback window (Figure 1). The next trial began automatically following the feedback window. The response window remained on-screen until the participant responded. During training, participants were offered an optional screen break every 20 trials to reduce fatigue.

All participants completed a total of ten training blocks of the motion discrimination task. Each day two training blocks were delivered in a single session over five consecutive days (2 training blocks of 400 trials per day, each lasting around 10 min) with a break of 1–2 min between training blocks carried out on the same day. Learning effect was quantified from additional assessment blocks on day 1 and day 5, which acted as the dependent variable (400 trials per assessment, each block lasting around 20 min). In these assessment blocks, stimuli were presented to the left or right visual hemifield in a pseudorandomly interleaved manner, with 200 trials per hemifield. For the training blocks, the stimulus was delivered to the right visual hemifield only, to allow the left hemifield to act as a control (i.e., contrast trained > untrained hemifield).

Task difficulty was adaptively modulated by altering the ratio of coherently moving dots to randomly moving dots, using a two up one down staircase procedure, described in detail elsewhere (Garcia-Perez, 1998). New staircases were initiated for every assessment and training block. For the assessment blocks, independent staircases were applied for the two visual hemifields. Motion direction discrimination thresholds for every block were calculated by taking the mean of the coherence on each reversal trial (the task changed from increasing in difficulty to decreasing, or vice versa). The first 10 reversals were discarded. The average provided a threshold at which the participant is predicted statistically to be correct 80% of the time.

### Statistical Analysis

The assessment sessions undertaken before and after training were used to measure the change in motion coherence thresholds. To quantify this change in discrimination threshold between the two assessments, a learning index was calculated, using the following formula:

$$\mathrm{LearningIndex}=\frac{\left(T_{1}-T_{2}\right)}{\left(T_{1}+T_{2}\right)}$$

where T_1_ and T_2_ are the thresholds for the first assessment and second assessment, respectively (Larcombe et al., 2017a,b).

A one-way analysis of variance (ANOVA) was used to determine whether the learning index (i.e., change in performance between the two assessments) differed between the three intervention groups (anodal tDCS, sham tDCS and no stimulation). Change in training performance was quantified using a two-way ANOVA with training block and intervention group as main variables.

In addition to the frequentist statistical approaches, a Bayesian repeated measures ANOVA was performed, using the open-source software package JASP^1^ (Wagenmakers et al., 2018a). Bayesian analyses permit a test of the relative strength of evidence for the null hypothesis (H_0_: no effect of tDCS stimulation group) versus the alternative hypothesis (H_1_: change in behavior as a result of tDCS condition) (Wagenmakers et al., 2018b). The equivalent one-way ANOVA on assessment data, and two-way ANOVA on training data were performed in JASP.

### Brain Stimulation

Participants received five sessions (20 min each) of tDCS delivered over left hMT+ (HDCkit, Magstim), one each day, concurrent with two 10-min training blocks. For sham stimulation the current was ramped up to 1 mA over 10 s and then switched off. For anodal stimulation, the current was ramped up over a duration of 10 s and remained at 1 mA for 20 min. Direct current was delivered through electrodes inside rectangular saline-soaked sponges. The cathode (8.5 × 6 cm) was placed at the vertex and the anode (5 × 5 cm) was placed 3 cm above the inion along the nasion-inion line and 6 cm left of the midline in the sagittal plane. The latter scalp coordinates were derived from prior research with transcranial magnetic stimulation (TMS), which showed effects of stimulation at this location on visual motion processing (Walsh et al., 1998; Hotson and Anand, 1999). The electrode montage used here has been used in previous tDCS research to stimulate left hMT+ (Antal et al., 2004a).

The experimenter who conducted the training and stimulation was blinded as to whether the participant was receiving sham or anodal stimulation. This was done using an automatic blinding mode on the tDCS stimulation device. Unblinding was performed once data collection was completed, prior to analysis. No blinding control was administered during the study.

## Results

**FIGURE 2:**
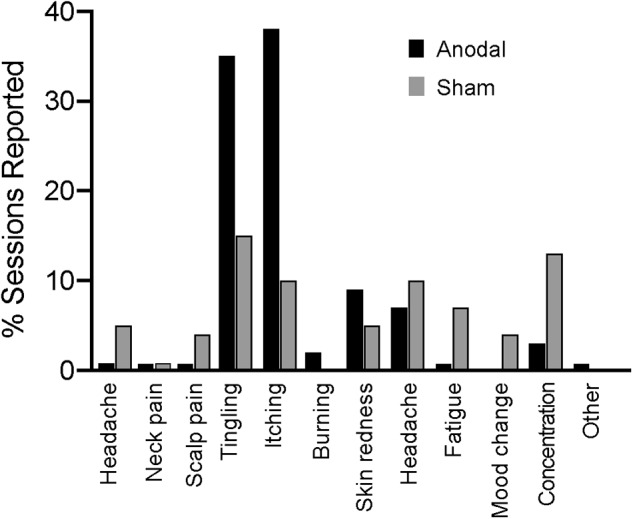
Adverse effects of stimulation as reported by participants.

There were no reported serious adverse effects in either group. Figure 2 shows all the reported effects of stimulation, of which itching and tingling were the most frequent.

Data from ten participants in a previous study (5 female, 18–29 years) using the same protocol, but without any stimulation, were included in the analysis for comparison (Larcombe et al., 2017b). For all assessment and training sessions, performance was quantified by determining the motion direction discrimination threshold, a measure used in previous studies to quantify changes in learning (Larcombe et al., 2017a,b).

**FIGURE 3:**
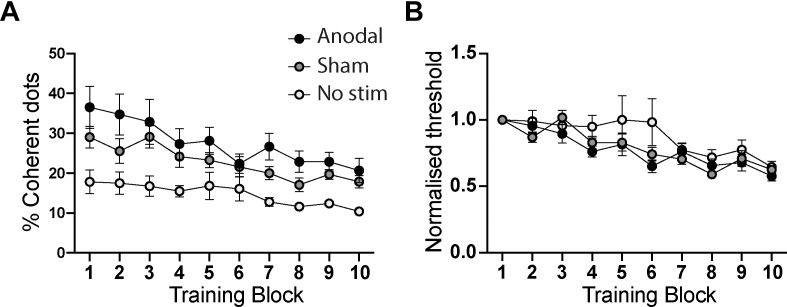
**(A)** Comparison of performance of anodal, sham and no tDCS stimulation groups across the ten training blocks. There is a significant effect of training and stimulation group, driven by the superior performance of the “no stimulation” group at baseline (training block 1). **(B)** shows the training performance normalized to the first training block, to quantify learning curves for each group while controlling for each individual’s differing baseline performance level. While the main effect of training remained significant, there was no effect of stimulation group nor an interaction between training and stimulation group. Error bars show ± SEM.

For training sessions, two analyses were performed, one using the raw coherence thresholds, and a second in which the thresholds were normalized within each participant relative to performance in the initial training block (i.e., block 1 of Day 1). The raw values are shown in Figure 3A for each of the training groups. There was a significant effect of Training Block, reflecting the improvement over time [*F*(9,252) = 17.9; *p* < 0.0001]. There was also an effect of stimulation group [*F*(2,28) = 6.4; *p* = 0.005], reflecting the considerably lower starting threshold of participants in the no stimulation group. The interaction, however, was not significant [*F*(18,252) = 1.6; *p* = 0.06].

There was no difference in threshold in the first training block between the two groups that were randomized to sham or anodal tDCS (independent samples *t*-test: *t* = 2.7; *p* = 0.12). There was a difference in this measure at baseline if all three groups are compared, but this simply reflects the lower starting threshold in the no stimulation group in the previous study [one-way ANOVA: *F*(2,30) = 5.7; *p* = 0.008].

Performance levels in the daily motion perception training blocks when normalized to the first block were indistinguishable across the three groups (Figure 3B). While there was a significant effect of training block [*F*(9,252) = 16.3; *p* < 0.001], indicating that participants learned the task, there was no difference between the anodal, sham and no stimulation groups [*F*(2,28) = 1.5; *p* = 0.23]. The interaction term, which would indicate a differential rate of learning between the groups, was also not significant [*F*(18,252) = 1.2; *p* = 0.24].

To aid interpretation of the null effect of tDCS on the normalized data, a Bayesian repeated measures ANOVA was also performed. The pattern of results was consistent across both frequentist and Bayesian analyses. The main effect of training was significant, reflected in a higher Bayes factor for the alternative hypothesis (H_1_: training changes behavioral performance) than the null hypothesis (H_0_: no behavioral effect of training; BF_10_ = 1.6 × 10^18^). In contrast, the Bayes factor for the effect of tDCS stimulation condition (H_1_) was less than one (BF_10_ = 0.37). The reciprocal value (BF_01_ = 2.7) suggests that the data are 2.7 times more likely under the null hypothesis (that there is no effect of tDCS condition) than the alternative hypothesis, providing anecdotal evidence for this conclusion. The Bayes factor for the interaction term is 0.13, providing strong evidence for no interaction (7.6 times more likely than the alternative hypothesis).

**FIGURE 4:**
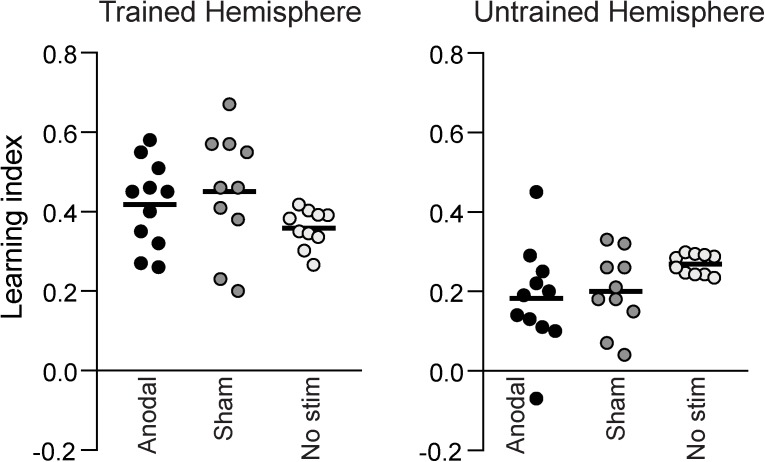
The learning indices for all participants, measured as the change between the pre- and post-training assessment sessions. There was no difference in learning index between the Anodal, Sham or No stimulation groups, neither in the hemisphere that was trained, nor in the untrained hemisphere.

Figure 4 shows the learning index calculated from the two pre- and post-training assessment sessions for the different groups. There was no significant difference between anodal, sham and no stimulation groups neither for the trained hemifield [one-way ANOVA: *F*(2,30) = 1.754, *p* = 0.192] nor the untrained hemifield [one-way ANOVA: *F*(2,30) = 2.283, *p* = 0.121]. A one-way Bayesian ANOVA was also performed on the learning index, and, consistent with the previous result, provided anecdotal evidence in favor of the null hypothesis (BF_10_ = 0.63; BF_01_ = 1.59). The effect sizes of the training-related task improvement for the sham and anodal groups were *d* = 4.3 and *d* = 3.0, respectively. The effect size for the difference between anodal and sham tDCS was small at *d* = 0.13.

Next we tested if anodal tDCS would enhance consolidation of visual learning across consecutive days. Offline consolidation refers to performance gains that occur after training during a rest interval. In this task, offline consolidation would be reflected in a lower direction discrimination threshold the day after training compared to the threshold achieved at the end of the previous day. Forgetting would be reflected in a threshold increase. Maintenance of learning would be reflected in no change across the interval between days. Figure 3 indicates there was no clear evidence of offline consolidation across consecutive days. Further, a one-way ANOVA on the mean difference in performance between consecutive days indicated no effect of tDCS on consolidation [*F*(2,30) = 1.52, *p* = 0.24]. Similarly, the Bayesian ANOVA provided anecdotal evidence in favor of the null hypothesis (BF_10_ = 0.54; BF_01_ = 1.85). We acknowledge that this measure of “consolidation” is not pure, however, since participants received tDCS (anodal or sham) during both blocks. A pure measure of consolidation would require a separate assessment session in the absence of stimulation.

## Discussion

All participant groups included in this study showed significant improvement in direction discrimination thresholds over the 5-day training period, consistent with previous results (Larcombe et al., 2017a,b). Furthermore, daily anodal tDCS to hMT+ during training had no effect on learning or offline consolidation.

All groups showed improved thresholds, i.e., learned from training. Yet, despite using stimulation parameters closely similar to previous tDCS studies of hMT+ (Antal et al., 2004b), there was no difference in performance between groups receiving anodal or sham tDCS. The improvement with training in both these groups was comparable to previous data from participants that had not received stimulation (Figure 3). There are several potential reasons for the lack of a tDCS learning or consolidation enhancement effect.

The current study differs from the majority of previous studies in healthy participants in that the stimulation was applied over a period of 5 days, rather than a single session. Since the tDCS protocol used here was based on these studies, differences are likely to be due to differences in the behavioral paradigm. One previous study was designed to investigate the effects of tDCS on the motion aftereffect, which is self-reported, rather than being a forced choice task, and anodal and cathodal stimulation had similar effects (Antal et al., 2004b). A second study by the same group found that anodal tDCS improved the percept of motion direction without distractors present, whereas cathodal tDCS improved the ability to determine direction of motion in the presence of distractors (Antal et al., 2004a). Indeed, a more recent study provided further evidence for an improvement in motion perception during tDCS over hMT+/V5, for both anodal and cathodal stimulation, although in different ways (Battaglini et al., 2017). The major difference between these previous studies and the current one is that our study aimed to test for an enhancement of learning (over multiple sessions), rather than a simple within-session change in behavior (no learning).

hMT+ was not identified in each participant individually using fMRI, and it is possible that the anodal electrode did not effectively stimulate the target area. However, this seems unlikely. Area hMT+ has been shown to vary only by approximately 2.7 cm in the left hemisphere (Watson et al., 1993) and to be, on average, 0.3 cm^3^ in size (Malikovic et al., 2007). The tDCS electrode dimensions exceed this (hMT+ anode: 5 × 5 cm), so it is likely that the stimulation at least partially covered hMT+. A related point is that the stimulation is applied at the scalp, and the achieved current dose within cortex is likely to vary across participants. The location of hMT+ is also variable across individuals, and can be either on a gyrus, in the sulcus, or both (Dumoulin et al., 2000; Large et al., 2016). How variations in individual anatomy interact with induced electrical current dose is currently under active investigation (Datta et al., 2012). Nevertheless, inter-participant variance in task performance was in a similar range for the anodal and sham groups, suggesting this is unlikely to be a key factor in the null result. Secondly, only the effects of anodal stimulation on a motion direction perception task were considered in this study. It may be interesting to investigate whether cathodal stimulation of hMT+ alters motion perception in this type of extended training protocol. Battaglini et al. (2017) found that both anodal and cathodal stimulation improved performance on a visual motion discrimination task, although the authors suggest the improvement was due to different mechanisms. We chose to stimulate with anodal tDCS as this polarity of stimulation has most reliably been associated with learning gains, at least in the motor system. A related point is the electrode montage and stimulation protocol (1 mA, 20 min) that was chosen, based on the majority of studies of the visual system, as reviewed by Antal and Paulus (2008).

A third point relates to the number of participants in the study. Variability in tDCS effects have led to calls for greatly increased sample sizes (Minarik et al., 2016). Our sample size (*n* = 10 or 11 per group) is comparable to several tDCS studies in the visual system that found significant effects (Walsh et al., 1998; Antal et al., 2004a) although it is smaller than a more recent study (*n* = 15) (Battaglini et al., 2017). The study was designed to test for a large effect of stimulation on learning, as has been reported for anodal tDCS to motor cortex during visuomotor learning (Reis et al., 2009; O’Shea et al., 2017). The null effects in our study do not exclude the possibility of a smaller effect that could be detected with a larger sample size.

One important, relatively neglected point in this discussion is that the end goal of much neuromodulation research is therapeutic. Here our motive for investigating tDCS was to advance the long-term goal of improving visual function in individual patients. For this to be practical, tDCS effects need to be measurable reliably in small samples, such as the single-case and small group designs that reflect the real-world challenges of clinical neuropsychology research and practice (Tikhonov et al., 2004). A small, but statistically significant effect that requires large populations to detect is unlikely to have measurable benefit at an individual level. Indeed, rehabilitation program for hemianopia that use similar training protocols to the one employed here (albeit for longer periods of time, e.g., 3 months) have effects sizes on the order of Cohen’s *d* > 2.5 (calculated from the data supplied in the published paper) (Cavanaugh and Huxlin, 2017). This reflects a consistent moderate improvement across participants on Humphrey visual fields, at a level considered clinically relevant in glaucoma patients (Tattersall et al., 2007). It is unknown to what extent adjunct brain stimulation might be expected to increase these effects.

Finally, multiple studies have shown that visual perceptual learning improves visual performance. We found no evidence that concurrent anodal tDCS to hMT+ accelerated perceptual learning or enhanced consolidation over a 5-day training period. It is possible that the training itself induced a ceiling effect in these young participants with a healthy visual system.

Although tDCS in these healthy participants did not improve visual motion discrimination, this does not rule out the possibility of a beneficial effect of the same intervention in a patient group. In healthy, sighted participants the main thalamocortical projection from the retina to V1 is intact. In contrast, patients with damage to the primary visual cortex must rely on other connections to convey retinal information to the visual cortex.

Since these alternative connections are unlikely to be as strong as the V1 pathway, it may be that training this pathway concurrent with electrical stimulation in patients would have a measurable effect.

## Conclusion

In conclusion, for the present study design, stimulation protocol and sample size, anodal stimulation over hMT+ in healthy participants during motion perception training did not improve performance compared to sham stimulation. This suggests that online, anodal stimulation of hMT+ (at least with the montage, current strength, duration, and participant sample tested here) may not be an effective way to modulate motion perception learning.

## Author Contributions

SL, CK, HB, and JO contributed to conception and design of the work. SL collected the data. SL and HB analyzed and interpreted the data. SL, HB, and JO drafted and critically revised the article. SL, CK, HB, and JO approved the final version to be published.

## Conflict of Interest Statement

The authors declare that the research was conducted in the absence of any commercial or financial relationships that could be construed as a potential conflict of interest.

## Footnotes

1 http://www.jasp-stats.org
